# Early Post-Discharge Suicide in Mental Health Patients: Findings From a National Clinical Survey

**DOI:** 10.3389/fpsyt.2020.00502

**Published:** 2020-06-09

**Authors:** Lana Bojanić, Isabelle M. Hunt, Alison Baird, Navneet Kapur, Louis Appleby, Pauline Turnbull

**Affiliations:** ^1^National Confidential Inquiry into Suicide and Safety in Mental Health, Faculty of Biology, Medicine and Health, The University of Manchester, Manchester, United Kingdom; ^2^Greater Manchester Mental Health NHS Foundation Trust, Manchester, United Kingdom

**Keywords:** follow-up, self-discharge, suicide method, personality disorder, mental health patients, post-discharge

## Abstract

Studies on suicide by recently discharged mental health patients have reported a high number of deaths in the early post-discharge period, which has led to recommendations of follow-up within 7 days (d). More recently, the National Confidential Inquiry into Suicide and Safety in Mental Health (NCISH) proposed a more “stringent” follow-up period of 2–3 days (d) after discharge. Patients who died within this early time-frame post-discharge were more likely to die before the follow-up appointment occurred. They more often had a primary diagnosis of a personality disorder, self-discharged, and had a higher frequency of death by jumping from a height or in front of the vehicle compared to later deaths. This study provides practical implications for post-discharge management and safety planning. Clinicians should be aware of (1) the increased risk of immediate suicide in the post-discharge period by people with a diagnosis of personality disorder, (2) immediate suicide risk in patients who initiate their own discharge, and (3) the increased risk of death by jumping from a height or in front of the vehicle in the immediate post-discharge period. Our findings support the recent recommendation from NCISH that follow-up should occur within 3 d of discharge from in-patient care.

## Introduction

Discharge from psychiatric in-patient care is a period of high risk for suicide (1). In the UK, post-discharge suicides make up 17% of all patient suicide deaths (2). A recent meta-analysis of suicide rates after discharge from psychiatric facilities suggests these rates remain high for several years (3), but are particularly high in the first few months (4, 5) and weeks (6–8) post-discharge. These findings have led to a recommended follow-up period within 7 days (d) of discharge for all patients, and within 48 h if a risk of suicide has been identified (9). There is some evidence that this intervention has impacted suicide rates; the 2018 report from the National Confidential Inquiry into Suicide and Safety in Mental Health (NCISH) (2) reported a fall in suicide rates within 3 months post-discharge in England following a peak in 2011.

However, as Chung and colleagues (1) point out, this time frame still means that some patients are likely to die before their scheduled appointment. In a controlled study of 100 patient deaths by suicide within 2 weeks of discharge, Bickley and colleagues (6) reported that 55% occurred in the first week, 49% of whom died before their first follow-up appointment. More recently, NCISH reported the second and third day post-discharge to be the highest risk periods and suggested a more “stringent” follow-up within 3 days (d) of discharge (2).

A number of studies have examined the characteristics of patients who died by suicide shortly after discharge. The majority have reported higher rates of post-discharge suicide in men (1), with the exception of a study linking a Finnish nationwide register with in-patient data (10) which reported more female patients dying in the first week post-discharge. Studies also report being older than 40 (6), unmarried (10), unemployed (11), and living alone or having low levels of social support (11–13) as risk factors for post-discharge suicide. In UK studies that have examined post-discharge suicide, the most common methods of suicide were hanging and self-poisoning (4–6). However, other international studies have reported more deaths by jumping from a height (12) and drowning (10), citing availability, and lethality of these methods. In respect of psychiatric diagnosis, post-discharge suicide is most commonly associated with diagnoses of affective disorders, namely depression, and schizophrenia (4, 10, 11). Examination of linked Swedish registers (14) found a higher risk of suicide within a month post-discharge in male patients with a diagnosis of depression and a reaction to crisis in comparison to patients with other diagnoses.

A finding repeated across studies regardless of the post-discharge time frame is a higher risk in patients who initiated their own discharge (4, 5, 7, 15–17). Further clinical factors associated with suicide include non-adherence to treatment (4, 10, 11), a short final admission (5, 6, 16, 18), and a history of/current self-harm (4, 12). Findings from prospective studies have shown that suicidal ideation was more elevated in patients who died by suicide 1, 3, and 6 months after discharge compared to other discharged inpatients (19, 20). However, little is known about whether the characteristics of patients who die by suicide within the immediate post-discharge period, i.e. less than a week, differ from later post-discharge deaths (2). An awareness of the features of these patients could aid care and discharge safety planning. In this study we aim to examine factors associated with immediate suicide following discharge from psychiatric in-patient care, and any subsequent practical implications for the management of care. We describe the socio-demographic and clinical characteristics of patients who died by suicide in the first 3 d after discharge in comparison with those who died between 4 and 7 d post-discharge, and present the rates of early post-discharge suicides.

## Materials and Methods

### Data Collection

NCISH collects data on all deaths by suicide by people in the United Kingdom who were in contact with mental health services in the 12 months prior to death (further referred to as “patients”). The method of the NCISH data collection is fully described elsewhere (21). In short, data collection starts with the identification of all cases of death by suicide including deaths with undetermined intent from the Office for National Statistics. Information on contact with mental health services in the 12 months before death is then obtained from the National Health Service (NHS) trusts and Health Boards in the deceased’s district of residence. Lastly, clinical data is obtained *via* questionnaires completed by the patient’s supervising clinician. The questionnaire collects information on socio-demographics, psychosocial history, life events, details of the suicide, treatment and adherence, and last contact with services (including the date of last discharge). The sample presented here consists of patients who died by suicide within a week of discharge from psychiatric in-patient care between January 1997 and December 2016 inclusive, based on the date of death. NCISH achieves a questionnaire response rate of >95% (4). NCISH has research ethics approval from the North West Research Ethical Committee and approval under Section 251 of the NHS Act 2006 (originally Section 60 of the Health and Social Care Act 2001).

### Statistical Analysis

**Figure 1:**
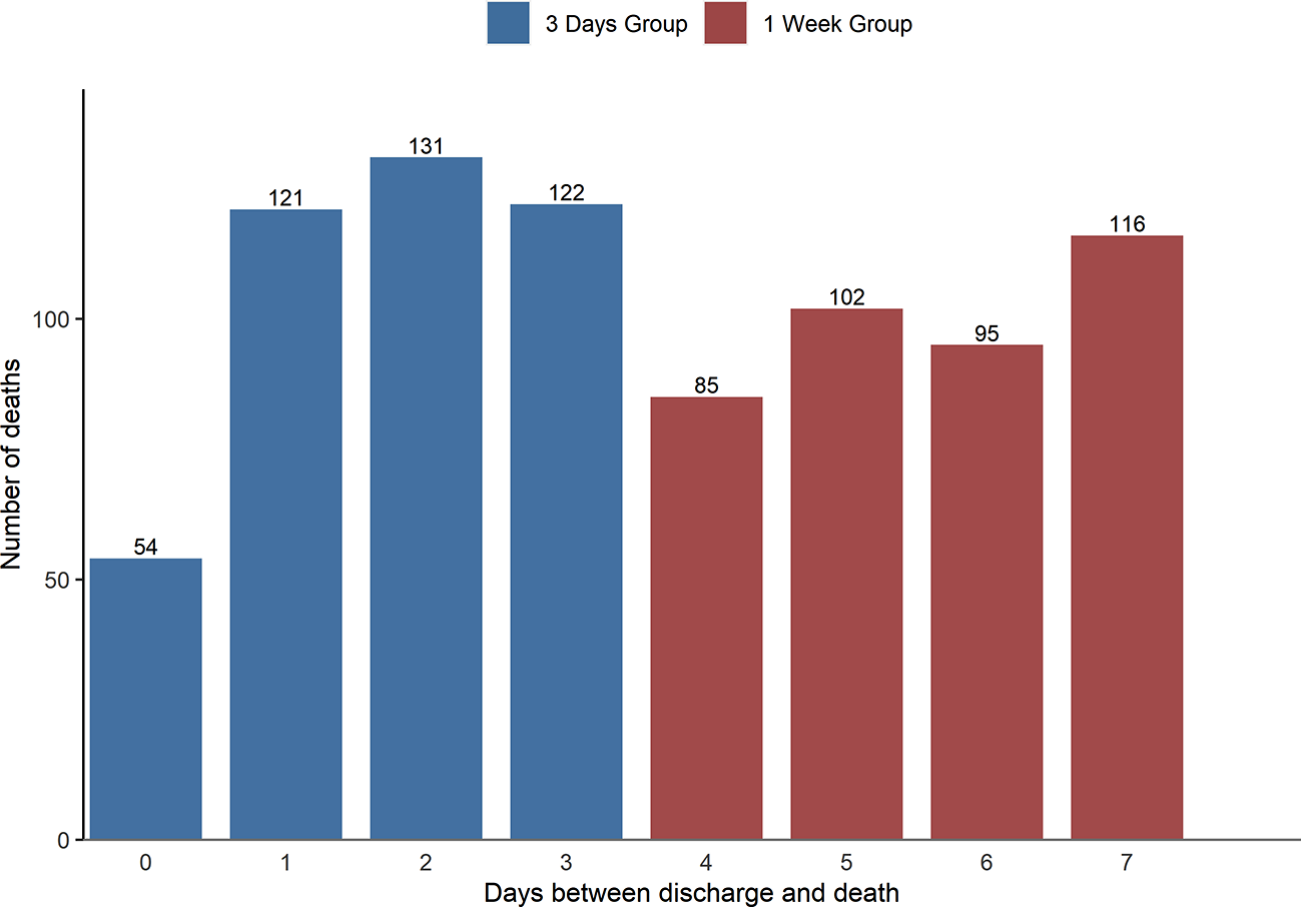
Frequency of deaths by suicide in England and Wales between 1st January 1997 until 31st December 2016 by discharge day (*N* = 826).

In the present analysis we focused on England and Wales data due to the differences in service provision between devolved nations. Two groups of patients were examined based on the time of their death in relation to discharge: patients who died by suicide within 3 d post-discharge (3 d group) and those who died by suicide between the fourth and seventh day post-discharge (1 week group). We grouped patients who died on day 0 post-discharge (i.e. on the actual day of discharge) into the 3 d group (**Figure 1**). We have only included community patients who have been formally discharged and had a discharge date recorded. Since we do not record time of the death and time of the discharge we have calculated the time between death and the discharge as the difference in days.

Descriptive analysis (presenting the frequency and percentages) and comparisons (using chi-square tests) between the discharge groups were carried out. Descriptive statistics are presented as valid percentages, adjusting for the occurrences of missing data. We compared patients on various socio-demographic and clinical characteristics, as well as on suicide method, primary diagnosis, and estimation of suicide risk by the clinician. Results with *p* < .05 were considered to be statistically significant.

Rates were calculated for the years with complete numerator and available denominator data from NHS Digital, i.e. 2005–2015. Data for 2016 were incomplete due to the time associated with legal processes and were therefore omitted from the rate analysis. Denominators used were total number of mental health in-patient admissions and discharges in England and Wales obtained from NHS Digital which collects data on in-patient activity. Rates for the 3 d group have been adjusted to account for the discrepancy between the number of days between groups. Due to the numbers per year being small, in-depth time series analysis could not be performed. This was also prohibitive in terms of adjusting for other demographic variables such as age and gender. All analyses were carried out using statistical software STATA Version 15 (22). All the figures were produced using RStudio 3.4.3 (23).

## Results

### Descriptive Analysis and Rates

Over the 20-year period from 1^st^ January 1997 until 31st December 2016, we were notified of 26,426 deaths identified as patient suicides (i.e. they had been in contact with mental health services in the 12 months before death). A total of 826 (3.1%) died within a week of discharge from psychiatric in-patient care. Within this time frame, 428 (51.8%) died within 3 d (3 d group) and 398 (48.2%) died between 4 and 7 d (1 week group). Patients in the two groups did not differ in age (median in the 3 d group=46 (range 16–94) v. median in the 1 week group = 46 (range 15–87), *χ²=*0.18, *df=*1, *p*=.67). Frequencies of suicide deaths by days since discharge are shown in **Figure 1**.

**Figure 2:**
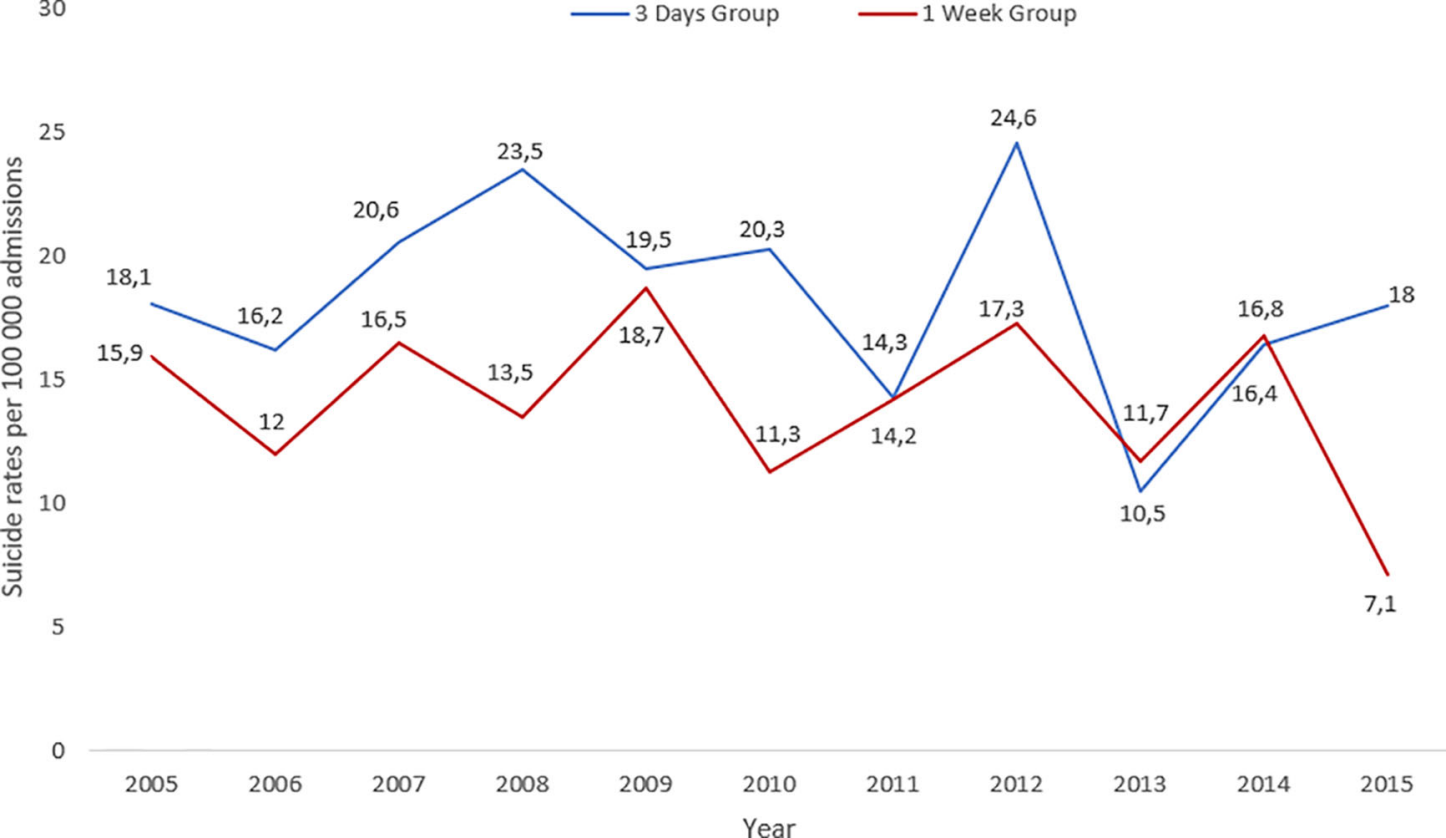
Rates of suicide per 100,000 admissions to psychiatric in-patient care per year in England and Wales between 1st January 1997 until 31st December 2015 for 3 d and 1 week group.

**Figure 3:**
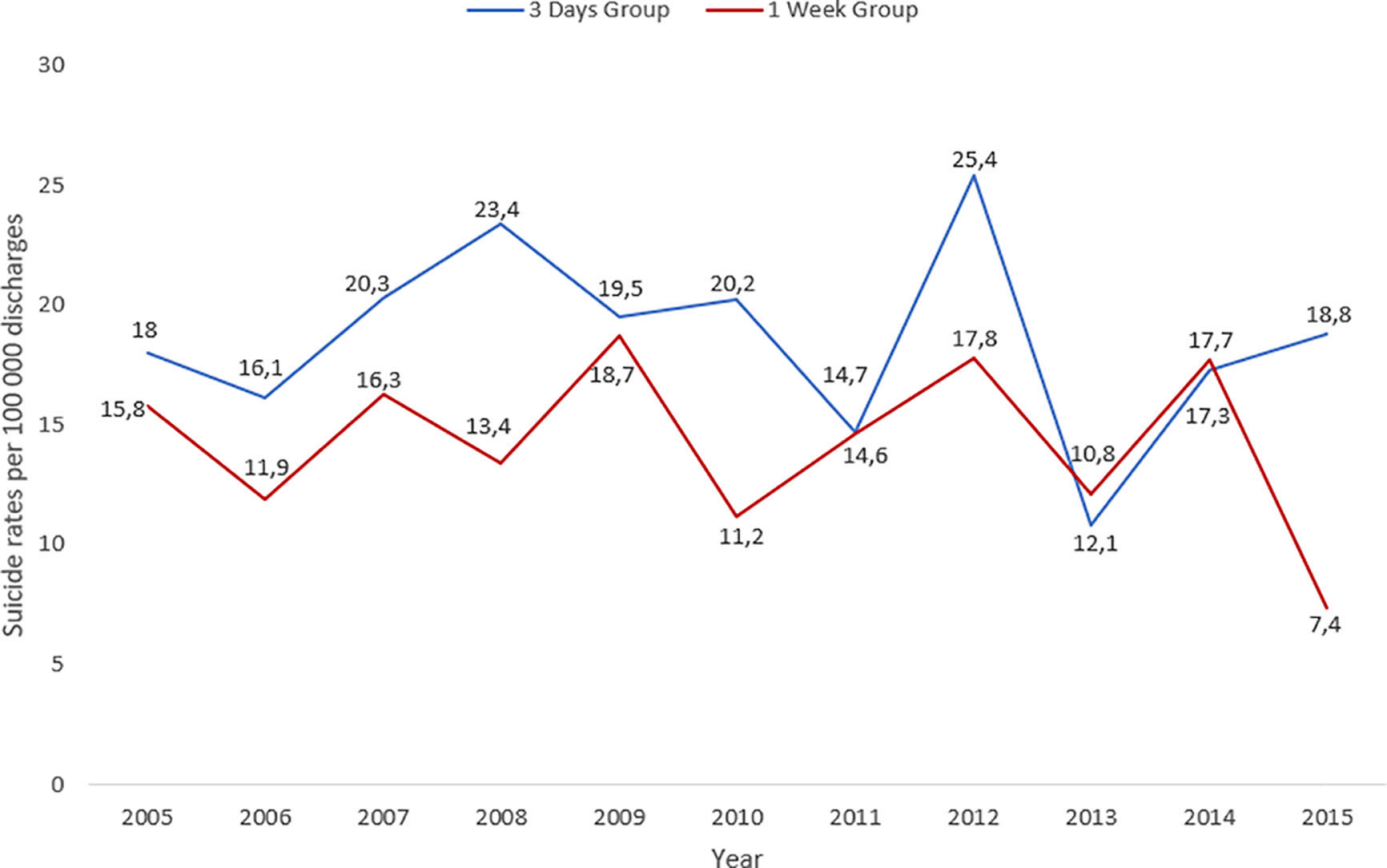
Rates of suicide per 100,000 discharges from psychiatric in-patient care per year in England and Wales between 1st January 1997 until 31st December 2015 for 3 d and 1 week group.

Early post-discharge suicide rates by year and number of admissions and discharges, respectively, are presented in **Figures 2** and **3**. Overall rates for the 3 d group were 0.18 per 100,000 admissions and 0.19 per 100,000 discharges. Overall rates for the 1 week group were 0.14 per 100,000 admissions and 0.14 per 100,000 discharges. Rates per admissions and discharges generally followed the same pattern. Despite fluctuations, rates were slightly higher for the 3 d group compared to the 1 week group.

### Comparisons Between Groups

Almost two-thirds of patients who died within 7 d of discharge were male, reflecting the ratio of men and women who die by suicide in the UK. There were no differences in the socio-demographic features between the two groups (**Table 1**).

**Table 1:** Sociodemographic and clinical characteristics of patients who died within a week post-discharge (*N* = 826).

Patient characteristic	3 Days Group *N* = 428 n (valid %)	1 Week Group *N* = 398 n (valid %)	*χ²*	*p*	
**Socio-demographics**					
Male	280 (65.4)	261 (65.6)	< .01	.96	
Black, Asian and minority ethnic group	20 (4.8)	22 (5.6)	.28	.60	
Unmarried	313 (73.8)	272 (68.9)	2.47	.12	
Unemployed	192 (45.0)	173 (44.5)	.02	.89	
Living alone	226 (53.1)	196 (49.9)	.83	.36	
Homeless	21 (5.0)	18 (4.6)	.05	.82	
**Suicide method**					
Hanging/strangulation	158 (36.9)	158 (39.7)	.68	.41	
Self-poisoning	79 (18.5)	87 (21.9)	1.49	.22	
Drowning	32 (7.5)	32 (8.0)	.09	.76	
Jumping from a height/in front of a vehicle	102 (23.8)	65 (16.3)	7.19	< .01**	
Other method^1^	40 (9.4)	34 (8.5)	.16	.69	
**Primary diagnosis**					
Schizophrenia and other delusional disorders	58 (13.6)	58 (14.6)	.15	.70	
Affective disorder (bipolar disorder & depression)	176 (41.5)	189 (47.6)	3.09	.08	
Substance dependence	50 (11.8)	40 (10.1)	.62	.43	
Personality disorder	65 (15.3)	37 (9.3)	6.81	< .01**	
Other primary diagnosis^2^	63 (14.9)	64 (16.1)	.25	.62	
Any secondary diagnosis	244 (57.6)	205 (51.8)	2.76	.10	
**Clinical characteristics**					
Duration of last admission <7 d	162 (38.2)	140 (35.3)	.76	.38	
Last admission was a re-admission	112 (27.0)	93 (23.5)	1.32	.25	
Last discharge was self-discharge	72 (17.1)	41 (10.5)	7.48	.02*	
Non-adherent to medication in last month prior to death	55 (13.1)	49 (12.8)	.02	.90	
Any adverse life event in preceding 3 months	219 (53.3)	205 (53.3)	< .01	.99	
No follow-up appointment arranged	49 (11.9)	29 (7.5)	4.38	.04*	
Follow-up arranged within 3 d post-discharge	157 (46.3)	116 (38.5)	3.94	.05	
Follow-up arranged within a week post-discharge	252 (74.3)	239 (79.4)	2.29	.13	
Suicide occurred before follow-up appointment	238 (64.2)	149 (41.1)	39.3	< .01**	
History of self-harm	309 (73.2)	293 (75.7)	.66	.42	
History of violence	93 (22.5)	82 (21.3)	.17	.68	
History of alcohol misuse	186 (44.2)	168 (43.1)	.10	.75	
History of drug misuse	126 (29.9)	117 (29.9)	< .01	.99	
**Estimation of suicide risk^3^**					
Immediate: low or none	328 (80.4)	304 (81.9)	.30	.58	
Long-term: low or none	164 (46.6)	169 (51.2)	1.46	.23	

*p < .05, **p < .01, ^1^other methods of suicide include firearms, cutting/stabbing, burning, electrocution, suffocation/asphyxiation, and CO poisoning. ^2^other primary diagnoses include adjustment disorder, anxiety disorders, organic disorders, dementia, eating disorders, ADHD/conduct disorder, somatoform/somatisation disorder, and pervasive development disorder. ^3^Estimation of risk was provided by the clinician filling out the questionnaire.

The most common primary diagnosis in both groups was affective disorder (bipolar disorder and depression). Those who died in the first 3 d were more likely than later post-discharge deaths to have a diagnosis of personality disorder. Patients that died within 3 d post-discharge and had a diagnosis of a personality disorder were also more likely to have an additional secondary diagnosis (19, 29.2% v. 46, 70.8%, *χ²=*5.49, *p=*.02), most commonly depressive illness (15, 32.6%). Clinical features were similar in both groups, with high rates of previous self-harm, recent adverse life events, and a short (< 7 d) last admission. However, those who died in the first 3 d were more likely than those who died later in the week to have initiated their own discharge. Further, patients in the 3 d group were more likely to die before the follow-up appointment (238, 64.2% v. 149, 41.1%, *χ²=*39.29, p < .01) or to have no follow-up appointment arranged (49, 11.9% v. 29, 7.5%, *χ²=*4.38, p=.04). For all patients who died following discharge, the most common method of suicide was hanging/strangulation. Nearly a quarter of patients in the 3 d group died by jumping from a height or in front of a moving vehicle, significantly more than later post-discharge deaths (23.8% v. 16.3%, *χ²=*7.19, *p* < .01).

## Discussion

### Main Findings

We found the characteristics of patients who died by suicide in the first few days after discharge to include a primary diagnosis of affective disorder (24), patient-initiated discharge (16), and hanging/strangulation as the most common suicide method (4). These fit well with the general characteristics of post-discharge suicides reported in other studies (1, 4, 10, 11).

It is important to note that patients in the 3 d group were more likely to die before follow-up compared to 1 week group. This implies that following a more stringent follow up time frame has the potential to save lives. A diagnosis of personality disorder was significantly more common in the 3 d group compared to patients in the 1 week group. The lack of clear pathways into care and referral options for patients with personality disorders may present an additional challenge in the care of these patients (25). This may be especially challenging at the time of discharge, both for patients and clinicians, and could lead to patients with personality disorder being discharged into services that may not be able to provide the specialist expertise recommended by the National Institute for Health and Care Excellence (NICE) guidance (25). The finding that patients in the 3 d group had initiated their own discharge more often than patients in the 1 week group is important. Self-discharge and a short final admission have been frequently cited in the literature as being associated with suicide (4, 5, 7, 15–17). Meehan and colleagues (5) acknowledged that patients who initiate their own discharge are by definition harder to engage with services after leaving hospital. A self-initiated discharge may indicate a lack of engagement with services, as well as more severe illness compared to patients who have been assessed as well-enough to transfer from in-patient to community care. Finally, patients in the 3 d group were more likely to die by jumping from a height or in front of a moving vehicle than those in the 1 week group. A previous study of NCISH data (26) found suicide by jumping a more common cause of death in inpatients and recently discharged patients than in the general population, hypothesizing the availability of the method and a lack of access to alternative methods as the most likely contributory factor. People who attempt suicide by jumping have often been described as ambivalent and if they are derailed from their attempt do not subsequently die by suicide (27).

On the whole, rates of patients who die in the early post-discharge period were small and fluctuated considerably over the study period. There was a peak in 2012, consistent with an overall increase in suicide by recently discharged patients that has since declined (2). In the last year with complete data (2015) there was an evident discrepancy between suicide rates in the 3 d and 1 week group, with the 1 week group showing a fall. However, due to low numbers per year, an in-depth analysis was not possible.

### Methodological Issues

This is, to the authors’ knowledge, the first study describing the characteristics of patients who died by suicide within two recommended follow-up periods post-discharge. However, there are some methodological limitations. As this is an exploratory, uncontrolled retrospective study, we cannot make causal inferences. Established limitations of NCISH data include the inability to draw etiological conclusions and the potential bias of the clinicians providing information (28). It is possible that some deaths by suicide may have been missed, however this is minimized by including deaths classified as both suicide and open verdict. Diagnoses were made by the treating clinician and we are unsure which tools were used during the diagnostic process. Of concern for this study in particular is the grouping of different types of personality disorders into one variable where the clinical picture for each disorder might differ. Additionally, the lack of information on the hour of patients’ discharge and death has prevented us from having a clearer picture of the time that had passed between the death and discharge and in return to place patients into post-discharge groups more precisely. For rates, numbers of post-discharge suicides per year were too small to allow for a meaningful time-series analysis. Finally, due to incompleteness, 2016 data could not be included in our rates analysis.

### Implications for Future Research

The comparison of patients who died by suicide in two recent post-discharge periods presents novel information with practical implications for discharge and safety planning. There is need for further research into discharge planning, both of a quantitative and qualitative nature. Specifically, more research is needed on the discharge needs of patients with different types of personality disorder (e.g. borderline, avoidant). Clinicians should be aware of the increased risk of suicide in the immediate post-discharge period by people with a diagnosis of personality disorder and in those who have initiated their own discharge, as well as the increased risk of jumping from a height or in front of vehicle shortly after discharge. This information should be incorporated into discharge and safety planning in collaboration with the patients and their families or carers where possible, including an assessment of the patient’s social support. Current UK guidance is for 7 d follow-up after discharge from in-patient care. The current findings suggest that deaths occurring immediately following discharge involve more highly lethal and immediately-available methods. Our findings support the recent recommendation from NCISH that follow-up should occur within 3 d of discharge from in-patient care (2). In addition, the data suggests this should be done as soon as possible—in some cases preferably on the very first post-discharge day.

## Data Availability Statement

The datasets generated for this study will not be made publicly available: Datasets contain personal details of patients that died by suicide.

## Ethics Statement

The studies involving human participants were reviewed and approved by North West Research Ethical Committee. Written informed consent for participation was not required for this study in accordance with the national legislation and the institutional requirements.

## Author Contributions

All authors contributed to the study conception and design. Material preparation and analysis were performed by LB. The first draft of the manuscript was written by LB and all authors commented on previous versions of the manuscript. All authors read and approved the final manuscript.

## Funding

This research was supported by the Healthcare Quality Improvement Partnership (HQIP NCA 2069, Mental Health Clinical Outcome Review Programme). The study was carried out as part of the National Confidential Inquiry into Suicide and Safety in Mental Health.

## Conflict of Interest

LA chairs the Suicide Prevention Advisory Group at the Department of Health and is a non-executive Director for the Care Quality Commission. NK is a member of the Suicide Prevention Advisory Group. NK also chaired the guideline development group for the 2012 NICE guidelines on the longer-term management of self-harm and currently chairs the guideline development group for the NICE depression in adults guideline.

The remaining authors declare that the research was conducted in the absence of any commercial or financial relationships that could be construed as a potential conflict of interest.
